# Identification of Feature Genes of a Novel Neural Network Model for Bladder Cancer

**DOI:** 10.3389/fgene.2022.912171

**Published:** 2022-06-01

**Authors:** Yongqing Zhang, Shan Hua, Qiheng Jiang, Zhiwen Xie, Lei Wu, Xinjie Wang, Fei Shi, Shengli Dong, Juntao Jiang

**Affiliations:** ^1^ Department of Urology, Shanghai General Hospital, Shanghai Jiao Tong University School of Medicine, Shanghai, China; ^2^ Department of Medicine, Shanghai General Hospital, Shanghai Jiao Tong University School of Medicine, Shanghai, China; ^3^ Department of Urology, Shanghai General Hospital, Nanjing Medical University School of Medicine, Shanghai, China; ^4^ Nursing Department, Shanghai General Hospital, Shanghai Jiao Tong University School of Medicine, Shanghai, China

**Keywords:** baldder cancer, neural network model, tumour-infiltrating immune cell, biomarkers, gene expression profiling, random forest

## Abstract

**Background:** The combination of deep learning methods and oncogenomics can provide an effective diagnostic method for malignant tumors; thus, we attempted to construct a reliable artificial neural network model as a novel diagnostic tool for Bladder cancer (BLCA).

**Methods:** Three expression profiling datasets (GSE61615, GSE65635, and GSE100926) were downloaded from the Gene Expression Omnibus (GEO) database. GSE61615 and GSE65635 were taken as the train group, while GSE100926 was set as the test group. Differentially expressed genes (DEGs) were filtered out based on the logFC and FDR values. We also performed Gene Ontology (GO) and Kyoto Encyclopedia of Genes and Genomes (KEGG) analyses to explore the biological functions of the DEGs. Consequently, we utilized a random forest algorithm to identify feature genes and further constructed a neural network model. The test group was given the same procedures to validate the reliability of the model. We also explored immune cells’ infiltration degree and correlation coefficients through the CiberSort algorithm and corrplot R package. The qRT–PCR assay was implemented to examine the expression level of the feature genes *in vitro*.

**Results:** A total of 265 DEGs were filtered out and significantly enriched in muscle system processes, collagen-containing and focal adhesion signaling pathways. Based on the random forest algorithm, we selected 14 feature genes to construct the neural network model. The area under the curve (AUC) of the training group was 0.950 (95% CI: 0.850–1.000), and the AUC of the test group was 0.667 (95% CI: 0.333–1.000). Besides, we observed significant differences in the content of immune infiltrating cells and the expression levels of the feature genes.

**Conclusion:** After repeated verification, our neural network model had clinical feasibility to identify bladder cancer patients and provided a potential target to improve the management of BLCA.

## Introduction

Bladder cancer ranks as the ninth most frequently diagnosed cancer and 13th in terms of deaths; this categorization causes more than 700,000 living cases and approximately 150,000 deaths per year worldwide (Antoni et al., 2017). Grading for the degree of risk of BLCA depends primarily on size, number, depth and differentiating degree of cancers (Bladder cancer, 2015). Approximately 10–34% of patients diagnosed with low-grade non-muscle-invasive BLCA die within 5 years. A range of factors can trigger the disease, such as occupational exposure to aromatic amines and hydrocarbons. Smoking is the most confirmed risk factor for bladder cancer. Bladder cancer is usually detected from painless gross haematuria symptoms and related imaging examinations, such as ultrasonography and computed tomography (Tsampoulas et al., 2008; Bladder cancer, 2015). In addition, nuclear matrix protein 22 (NMP22), bladder tumor antigen (BTA), fluorescence *in situ* hybridization (FISH) and other detection techniques have become screening tools for bladder cancer (Brausi et al., 2011; Wood, 2014).

Artificial neural networks are a family of machine learning models that are inspired by biological neural networks and are employed to estimate generally unknown functions (Jaddi et al., 2017). Related bioinformatic studies can be classified into five main categories: input modification, input reconstruction, saliency maps, convolution kernel analysis and attention mechanisms (Talukder et al., 2021). The artificial neural networks are mainly divided into three groups: feedforward neural network, recurrent neural network, and convolutional neural network depending on the different types of algorithms. The learning techniques can approximate functions and dynamics by learning from samples and have become powerful tools of deep learning and artificial intelligence (Kriegeskorte and Golan, 2019). In cancer genomics, neural network models are likely to be promising tools for extracting high-level features and learning prognostic information from multicancer datasets (Yousefi et al., 2017; Talukder et al., 2021). Various types of artificial neural network methods have been widely adopted in cancer research and provide accurate basic models for the early prediction of different cancers (Wan et al., 2019). However, ideal models including biomarkers that can make precise judgments with enough specificity and sensitivity for the diagnosis, treatment, postoperative follow-up and prognosis of BLCA are still lacking (Gofrit et al., 2006; Ng et al., 2021).

In this study, we attempted to construct a novel predictive artificial neural network model for the early diagnosis and evaluation of BLCA. Three expression profiling datasets downloaded from the GEO database were reanalyzed and feature genes selected from DEGs based on their importance scores were taken as candidate biomarkers. We also conducted qRT–PCR to measure the expression levels of these genes to demonstrate the accuracy of the model.

## Methods

### Downloading of Public Data

The GEO (https://www.ncbi.nlm.nih.gov/geo/) database is a public functional genomics data repository containing substantive high-throughput sequencing experimental data. Three expression profiling datasets (GSE61615, GSE65635, GSE100926) containing BLCA samples and normal para-tumor tissue were downloaded from the GEO expression array, and the genes expression matrix was organized for further statistical analysis (Zhao et al., 2015; Borisov et al., 2018; He et al., 2018). The statistical processing and graphic plotting were implemented using R software (R Version 4.1.2). We divided the expression profiling datasets into a train group containing GSE61615 and GSE65635, and a test group containing GSE100926.

### Identification of Differentially Expressed Genes

Bladder carcinoma and normal para-tumor tissue samples of the train group were set as the treatment and control groups respectively. The criteria were formulated as logFC ≥2 or ≤ −2 and *p* value <0.05 to filter out DEGs through the limma R package (Version 3.30.7). Then, we drew a heatmap and a volcano map to visualize DEGs through the pheatmap R package (Version 1.0.12) and ggplot2 R package (Version 3.1.0).

### Visualization of the Enrichment Analysis of Gene Expression Networks

Metascape (https://metascape.org/gp/index.html) was used for pathway and process enrichment analysis of DEGs. Based on their membership similarities, terms with a *p* value <0.01, a minimum count of three and an enrichment factor >1.5 were collected and grouped into clusters. We performed GO (http://www.geneontology.org/) and KEGG (http://www.genome.jp/kegg/) pathway analyses to investigate the physiological functions of the up- and down-regulated DEGs. We also constructed a protein–protein interaction (PPI) network using STRING website tools and Cytoscape software (Version 3.9.1) (Zhou et al., 2019).

### Screening and Scoring of Feature Genes

Based on the minimum point of cross-validation error in the random forest algorithm, those genes whose importance scores >0.30 were selected as feature genes associated with BLCA. Then, we drew a heatmap of these characteristic genes and scored the genes according to the following criteria. For those genes that were upregulated in tumor samples, if their expression levels were greater than the median value, one point was added to the scores. For those genes that were downregulated, one point was added to their scores when their expression levels were less than the median level. Then, we obtained the scoring list of the feature genes for further analysis.

### Construction and Validation of the Neural Network Model

The input layer of the novel neural network model was consisted of the scores and weights of feature genes. The hidden layer was constructed based on the feature information extracted from the input layer. Consequently, the function of the output layer was to judge whether the samples belonged to the control group or the treatment group. We also plotted the receiver operating characteristic (ROC) curve reflecting the predicted correctness rate of the model and calculated the area under the curve (AUC) values. We took the same measures to acquire the scores of feature genes in the test group (GSE100926), and a ROC curve with the AUC values was also incorporated into the validation criteria.

### Exploration of the Role of Immune Infiltrating Cells in the Neural Network Model

Evaluation of tumor infiltrating immune cells was calculated by using the CIBERSORT algorithm. Consequently, the correlation between different immune cell types was tested by Spearman (Rho) coefficients and presented as a correlation matrix. The results of differential analysis of immune cells between the control and treatment groups were examined by the Wilcoxon test and presented in the form of a violin diagram.

### Verification of the Expression of Feature Genes by qRT–PCR *in Vitro*

To further verify the neural network model, we performed qRT–PCR to analyze the relative expression level of the feature genes in human bladder urothelium cell (SVHUC-1) and urinary bladder transitional cell carcinoma samples (RT112 and T24) *in vitro*. RNA from Svhuc-1, RT112 and T24 cells was extracted with TRIzol (Novabio, China). Reverse transcription was implemented to obtain cDNA, and an ABI 7500 Real-Time PCR system (Applied Biosystems) was adopted to analyze the amplification of the cDNA. All the procedures were carried out three times independently and repeatedly.

## Results

### Acquisition and Grouping of Raw Expression Profiling Data

We downloaded three expression profiling datasets (GSE61615, GSE65635, GSE100926) from the GEO human gene expression array. The numbers of bladder tumor samples that the three datasets contained were, respectively, 2, 8, and 3, and the numbers of normal para-carcinoma tissue samples were, respectively, 2, 4, and 3. GSE61615 and GSE65635 were merged as a unified train group, and 17,891 genes expressed in their samples were incorporated into the subsequent analysis.

### Identification of Differentially Expressed Genes

**FIGURE 1:**
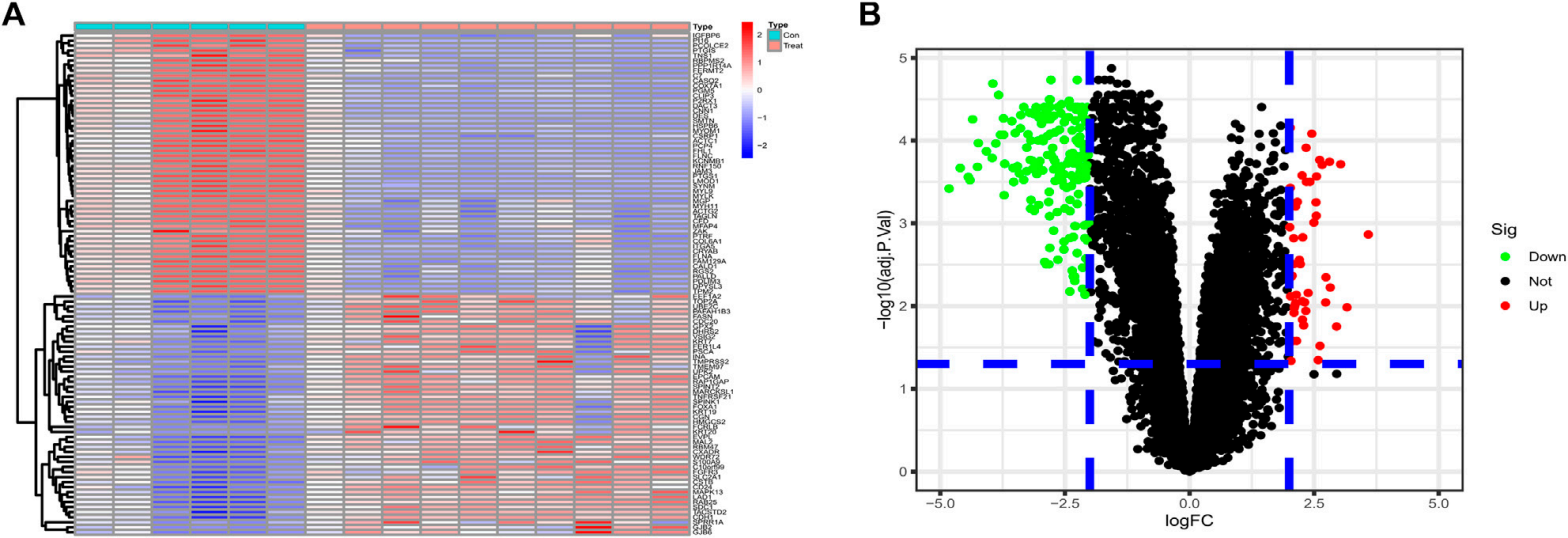
**(A)** The 50 DEGs most significantly upregulated and 50 most downregulated identified from datasets GSE61615 and GSE65635 were visualized in the form of a heatmap. **(B)** Based on the criteria logFC ≥2 or ≤ −2 and *p* value <0.05, 242 ponderable DEGs were filtered out and a volcano map was drawn.

Differential genes were screened according to the edgeR filter criteria (log2|fold change| > 2, FDR <0.05), and 242 ponderable DEGs between BLCA samples and normal para-carcinoma tissue samples were subsequently filtered out. Afterward, the 50 genes most significantly up- and down-regulated were visualized in the form of a heatmap (Figure 1A). We also drew a volcano map to present all filtered DEGs (Figure 1B).

### Visual Enrichment Analysis of Gene Expression Networks

**FIGURE 2:**
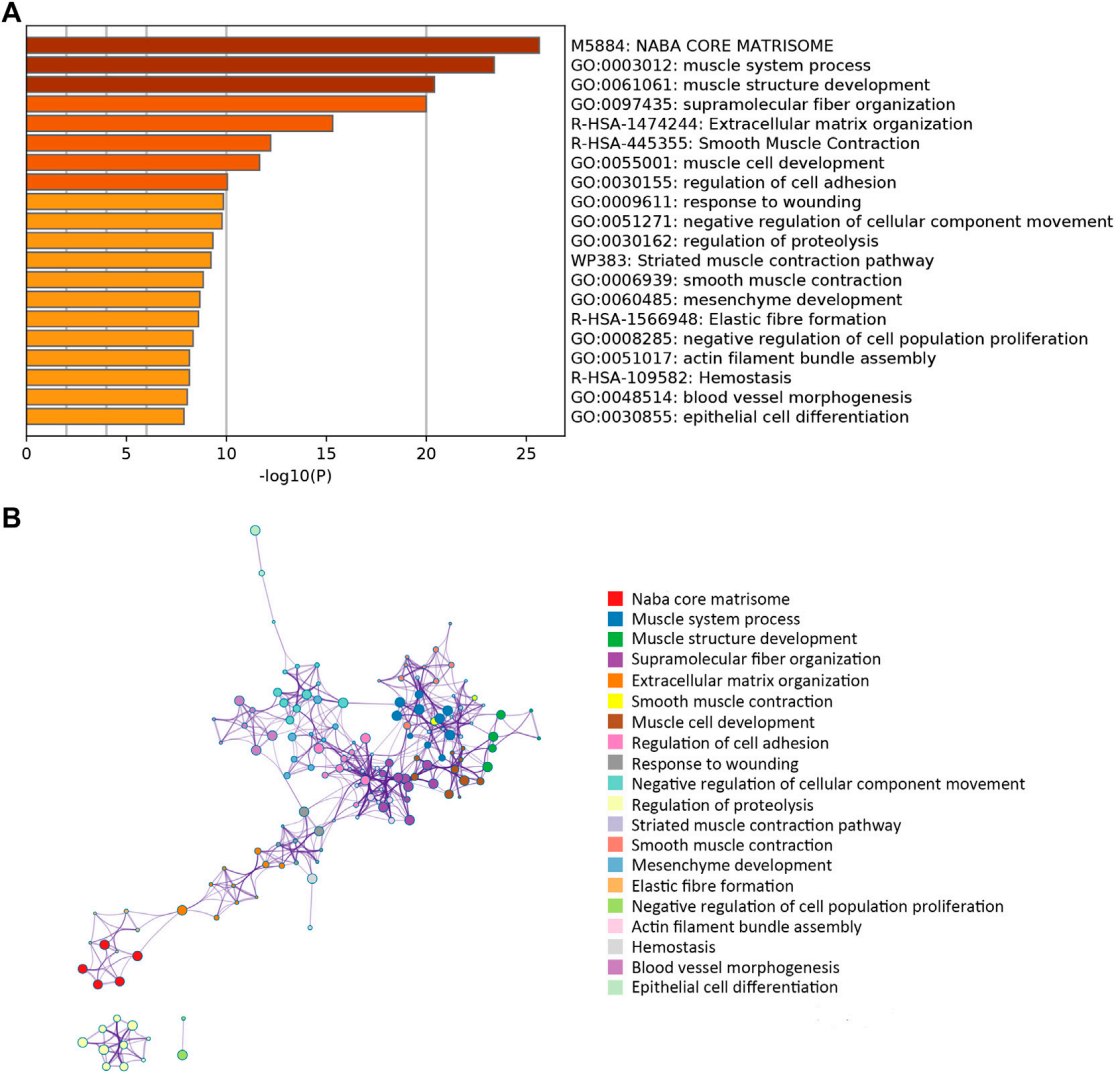
**(A)** Bar plot illustrating which functions or pathways DEGs were enriched by Metascape tools. **(B)** The term-enriched network was colored by cluster ID, where nodes sharing the same cluster ID are usually close to each other.

**FIGURE 3:**
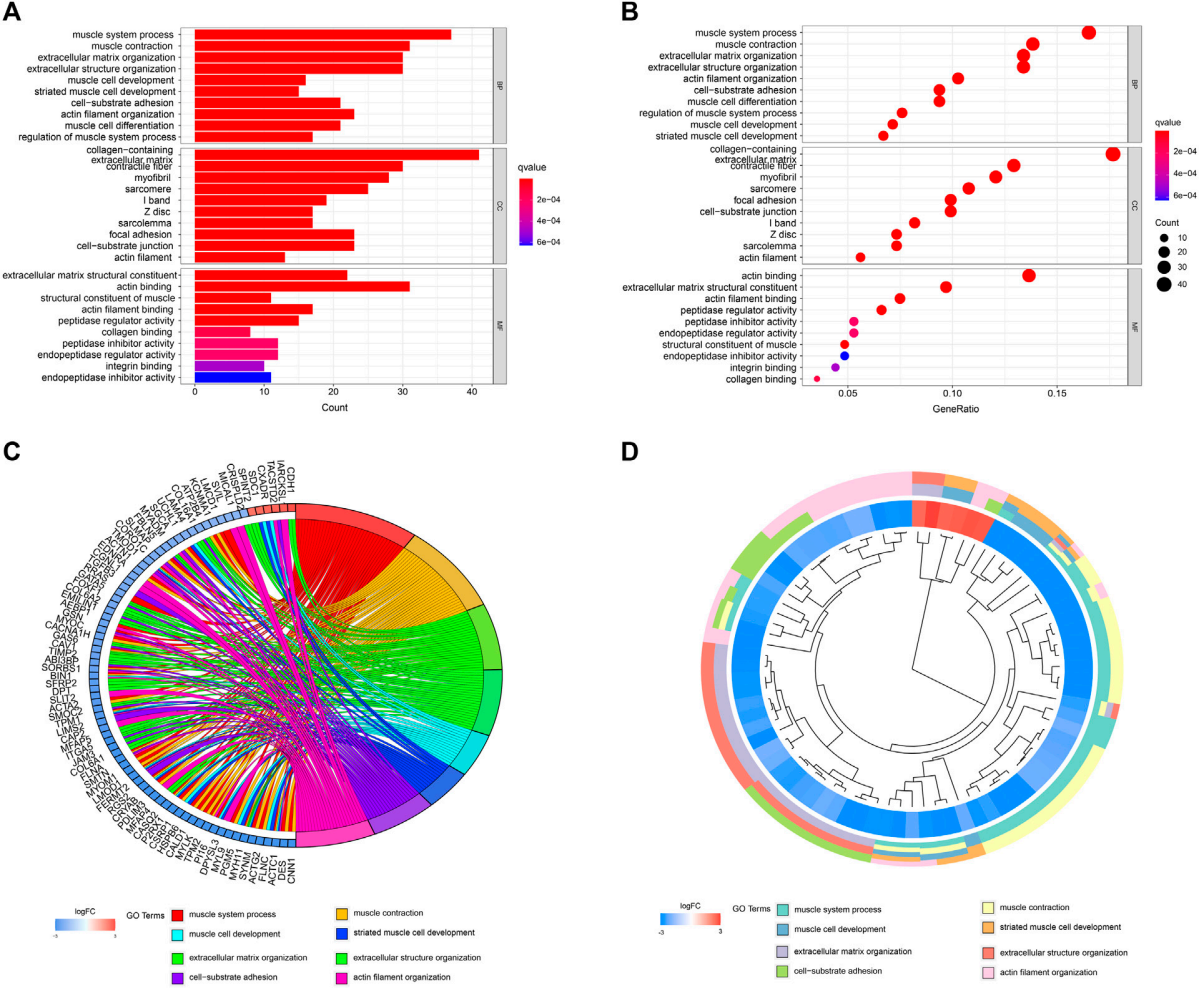
**(A,B)** Detailed information relating to changes in the biological processes (BP), cellular components (CC) and molecular functions (MF) of DEGs in control and treatment group through GO enrichment analysis. **(C,D)** Characteristic changes in up- and downregulated DEGs and their relationships with different BPs, CCs and MFs.

**FIGURE 4:**
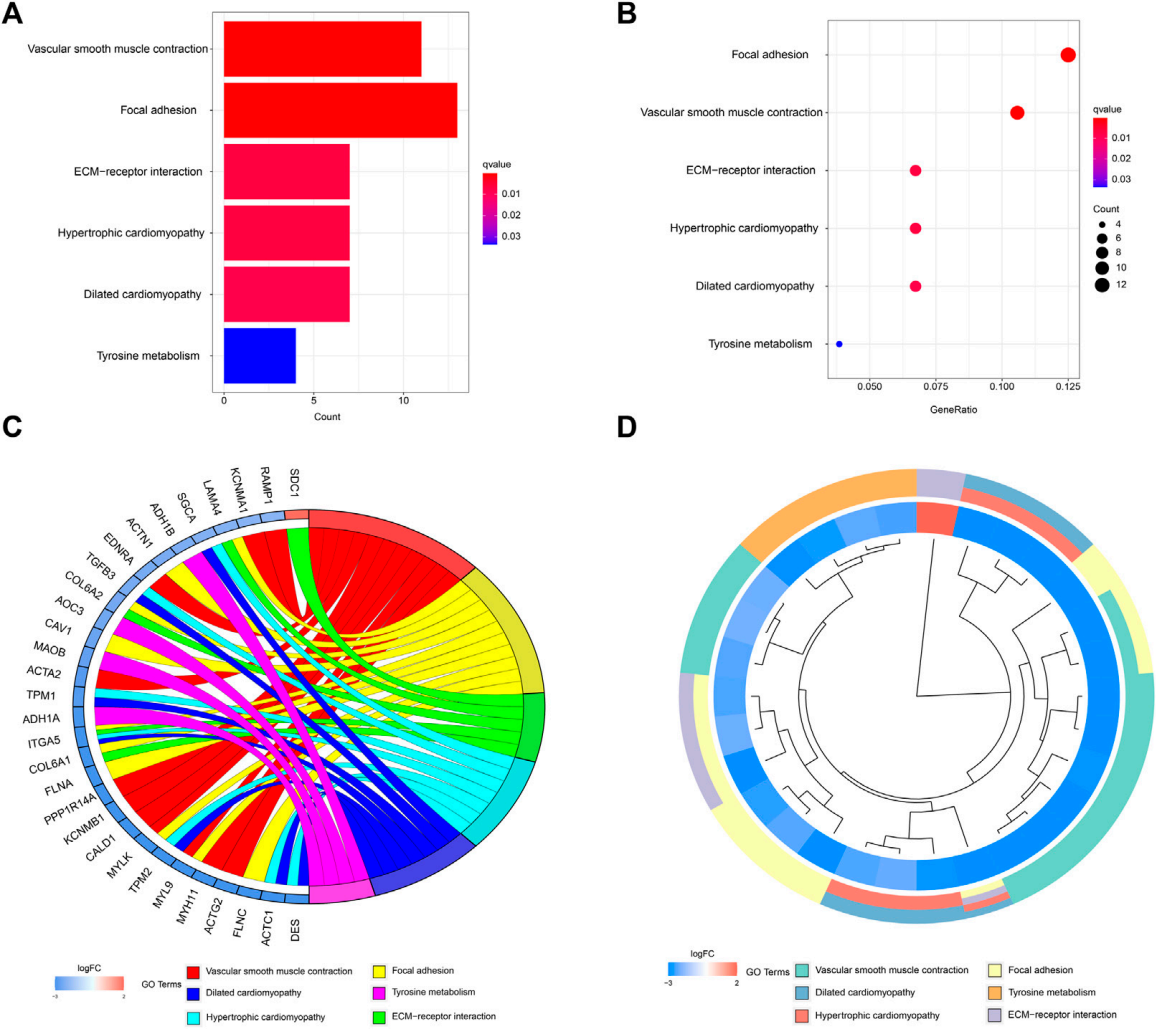
**(A,B)** Detailed information relating to changes in the functional pathways in the control and treatment group through KEGG pathway analysis. **(C,D)** characteristic changes in up- and downregulated DEGs and their relationships with different functional pathways.

**FIGURE 5:**
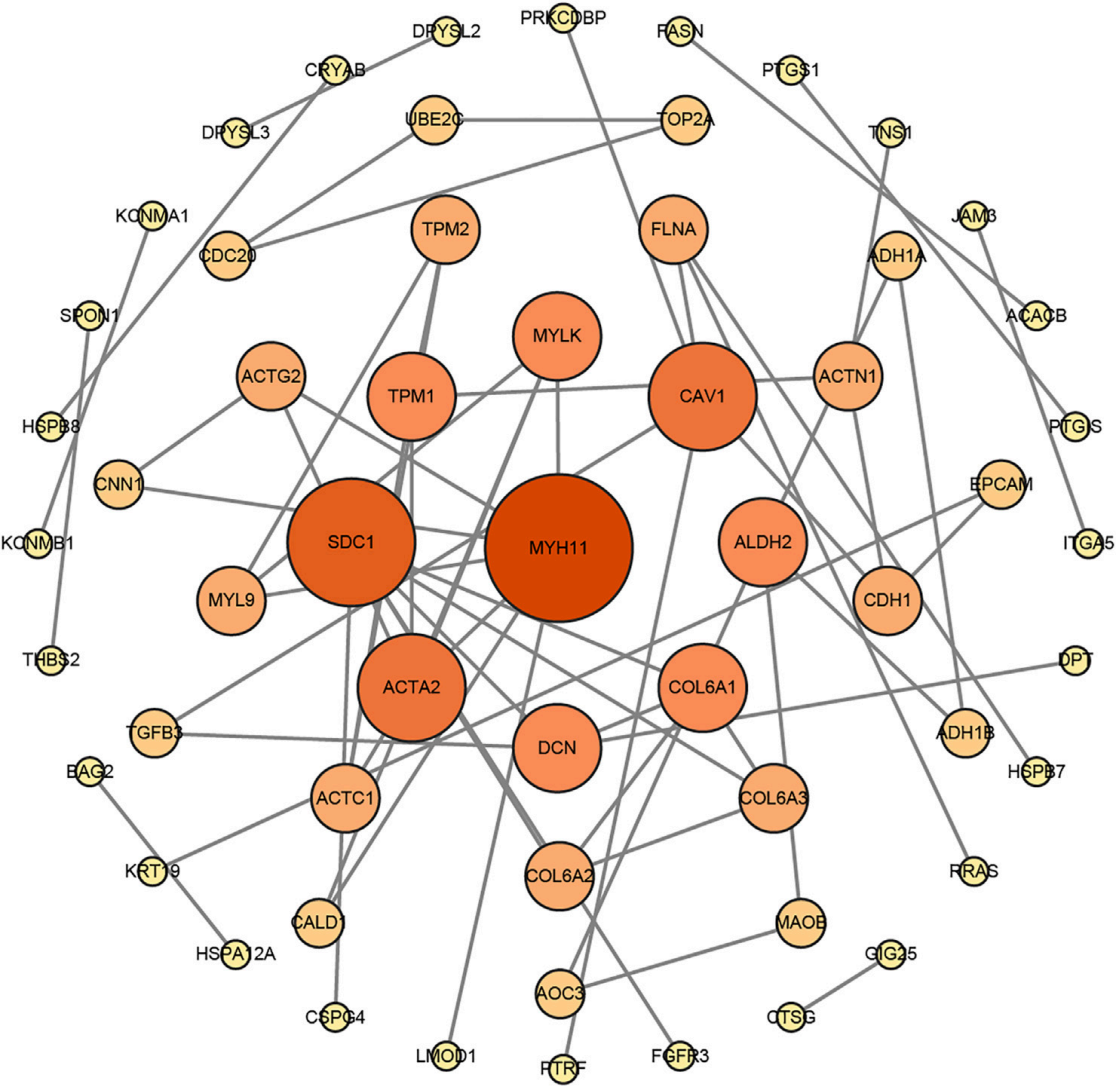
The protein–protein interaction (PPI) network of DEGs was constructed by the STRING website tools and modified by Cytoscape Software.

A network diagram (Figure 2A) and a bar diagram (Figure 2B) were drawn to show the results of the Metascape analysis. According to GO enrichment analysis, DEGs were significantly enriched in various biological processes, such as actin filament organization, extracellular structure organization, extracellular matrix organization and muscle cell development (Figures 3A–D). The results of the KEGG pathway analysis revealed that the DEGs were significantly enriched in pathways associated with vascular smooth muscle contraction, focal adhesion, ECM−receptor interaction and hypertrophic cardiomyopathy (Figures 4A–D). Afterward, we constructed a protein–protein interaction network to elucidate the intricate relevance of DEG-associated proteins (Figure 5).

### Screening and Scoring of Feature Genes

**FIGURE 6:**
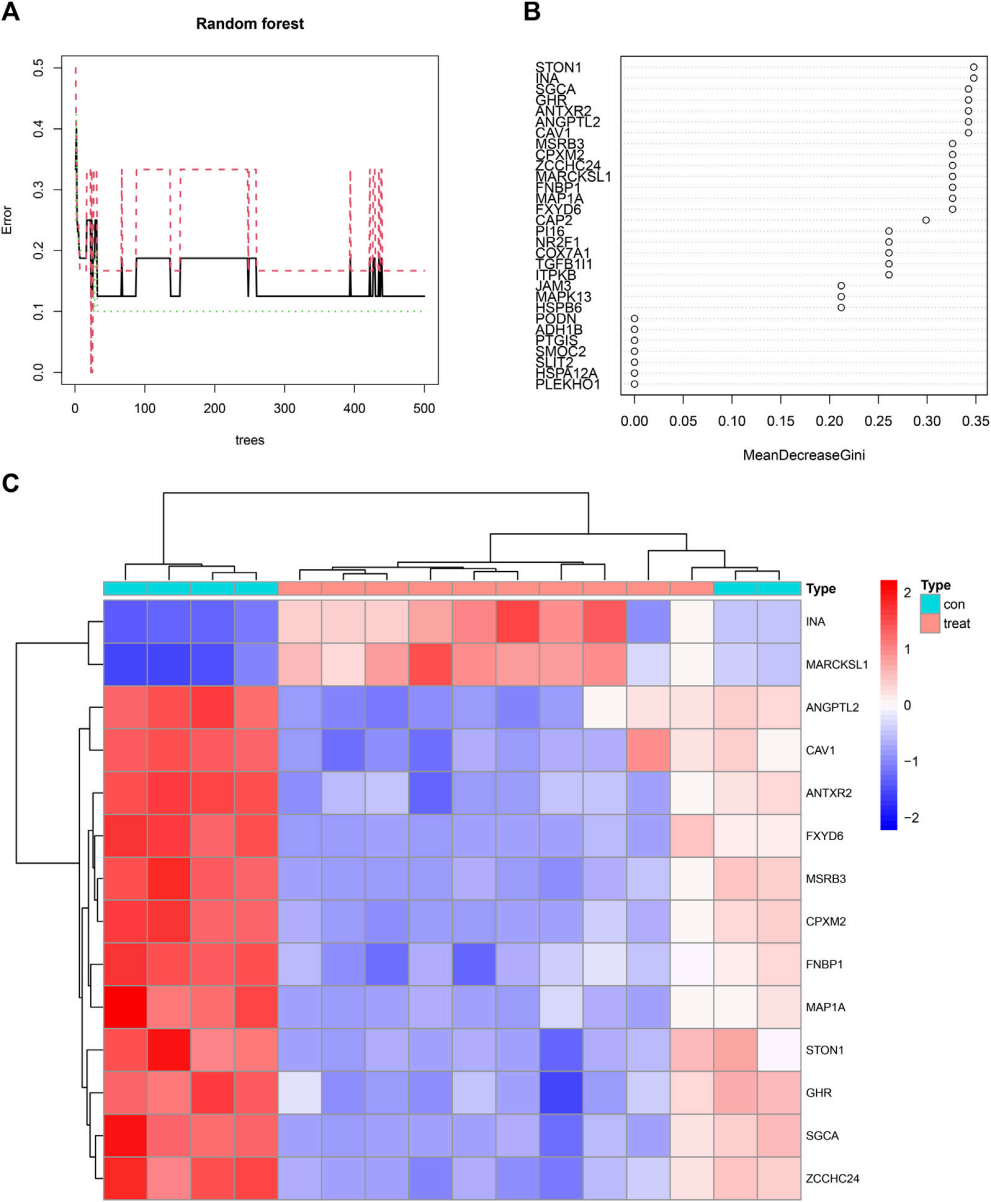
**(A)** A random forest map emphasizing the minimum point of cross-validation error in the process of filtering feature genes. The X-axis represented the number of trees and the Y-axis represented the error of cross-validation. The green curve represented the error of the treatment group. The red curve represented the error of the control group. The black curve represented the error of all samples. **(B)** Fourteen feature genes whose importance score was >0.30 were filtered out. **(C)** A heatmap illustrating the differences in the expression profiles of 14 feature genes between the control and treatment groups.

Fourteen feature genes whose importance score was >0.30 were filtered out based on the minimum point of cross-validation error in the random forest model (Figure 6A). They were STON1, INA, SGCA, GHR, ANTXR2, ANGPTL2, CAV1, MSRB3, CPXM2, ZCCHC24, MARCKSL1, FNBP1, MAP1A, and FXYD6 (Figure 6B). Differences in the expression levels of the 14 feature genes between the control group and the treatment group were presented as a heatmap (Figure 6C).

### Construction and Verification of the Neural Network Model

**FIGURE 7:**
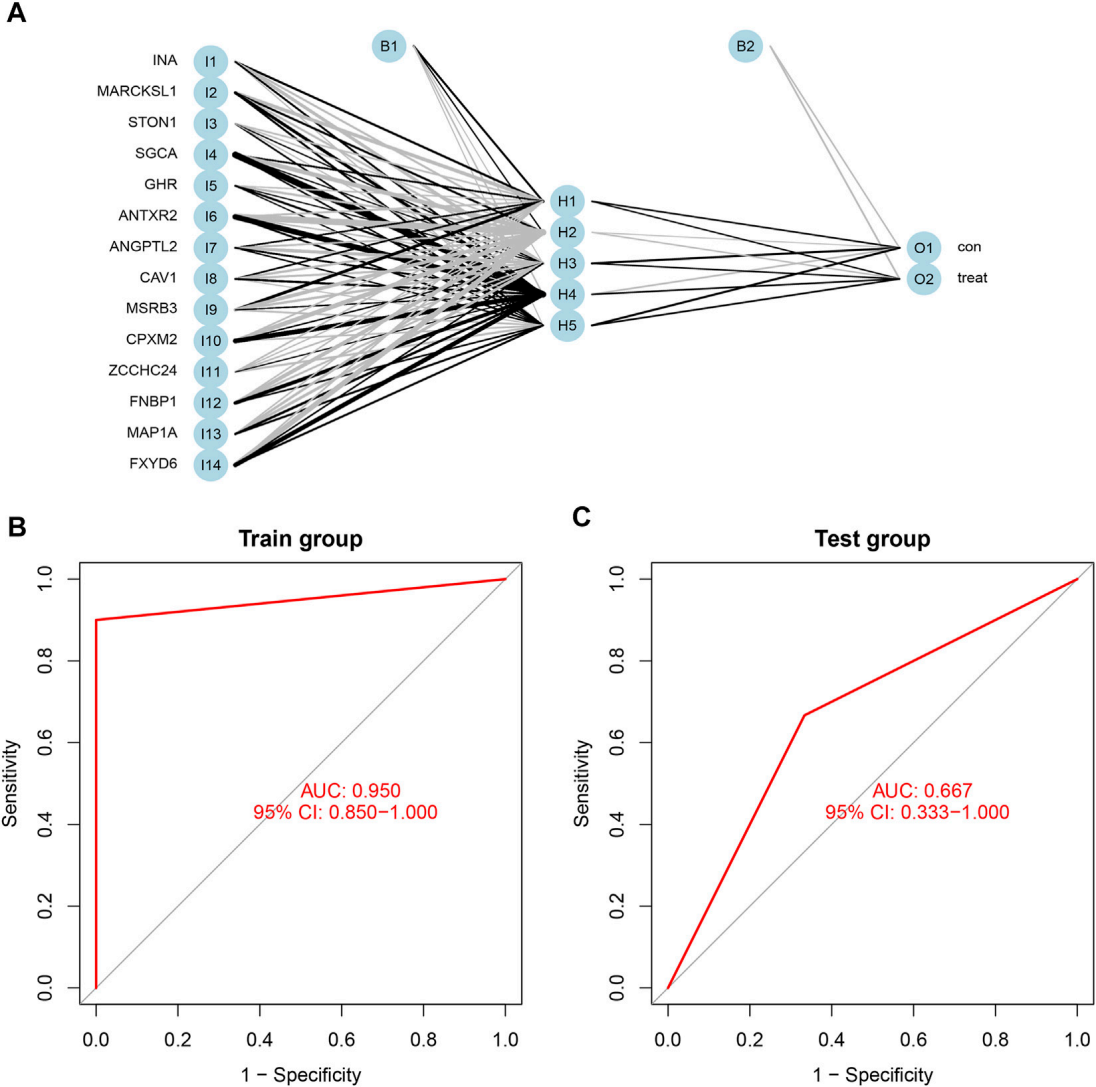
**(A)** The neural network model included three layers: input layer, hidden layer, and output layer. the scores and weights of feature genes. **(B)** The ROC curve of the train group including GSE61615 and GSE65635; the AUC was 0.950 (95% CI: 0.850–1.000). **(C)** The ROC curve of the test group including GSE100926; the AUC was 0.667 (95% CI: 0.667–1.000).

Based on the scores and weights of the feature gene list, we constructed a novel neural network model to predict whether the sample belonged to the control group or the treatment group (Figure 7A). All six samples in the control group were predicted correctly, and nine of the 10 samples in the treatment group were forecasted accurately. Afterward, we calculated the AUC value of the ROC curve, which was 0.950 (95% CI: 0.850–1.000) (Figure 7B). For further verification of the predictive accuracy of the model, the same measures were taken to acquire the scores of feature genes in the Test group (GSE100926). Two of three samples from both the control and treatment groups were predicted correctly, and the AUC value was 0.667 (95% CI: 0.333–1.000) (Figure 7C).

### Exploration of the Role of Immune Infiltrating Cells in the Neural Network Model

**FIGURE 8:**
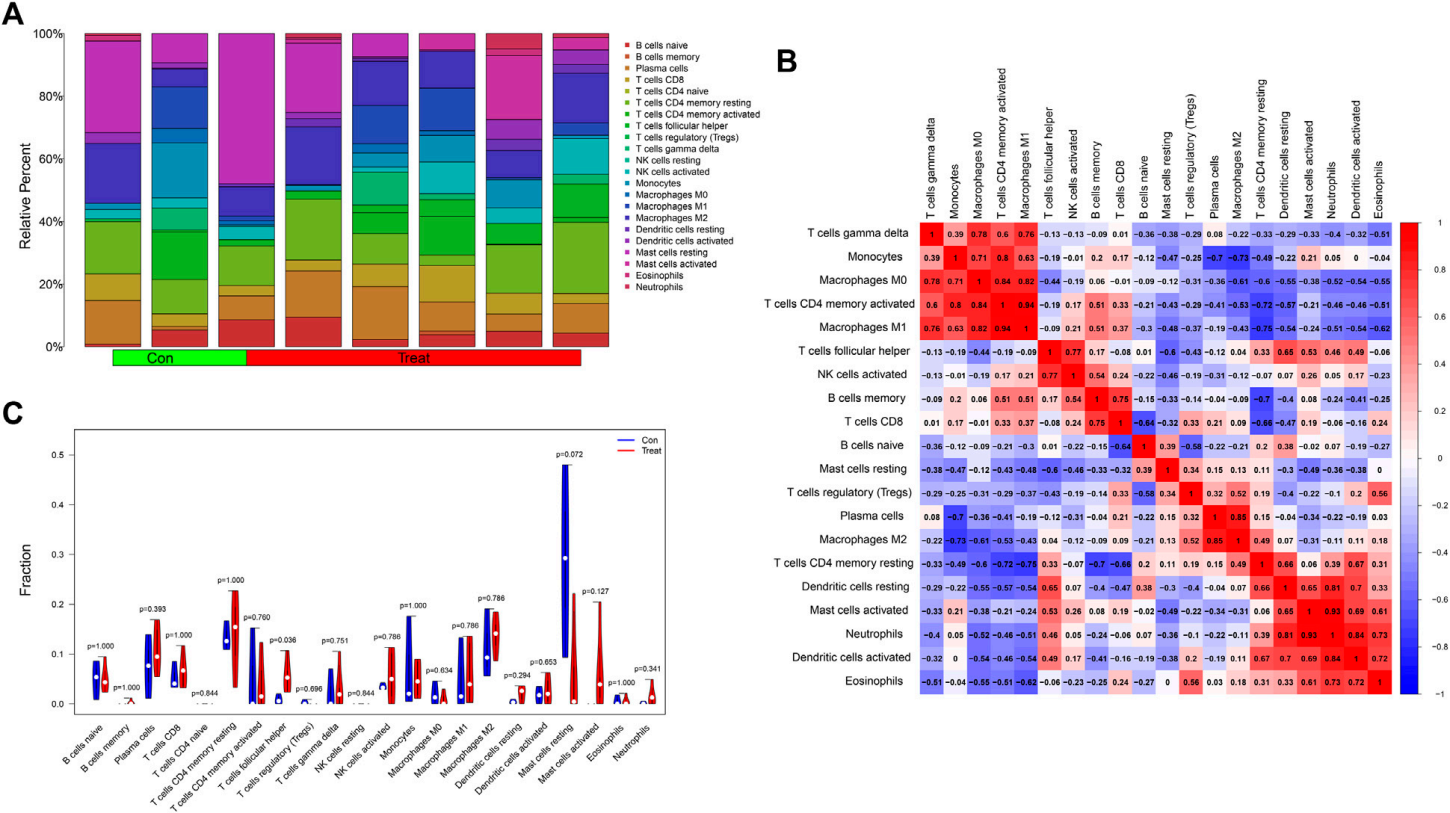
**(A)** A histogram showing the contents of 22 kinds of infiltrating immune cells in the samples with a threshold *p* value <0.05. **(B)** The heatmap showed the correlations between immune infiltrating cells. Red represented positively related while blue represented negatively related. **(C)** The differential analysis of immune cells was presented in the form of a violin diagram. The Y-axis represented the fractions of immune infiltrating cells.

We analyzed the contents of 22 kinds of immune infiltrating cells in the samples with a threshold *p* value <0.05 (Figure 8A) and further adapted a bar graph to visualize the positive and negative correlations between immune infiltrating cells (Figure 8B). According to differential analysis of immune cells between the control and treatment groups, we concluded that the content of follicular helper T cells was higher in the treatment group while resting mast cells was higher in the control group (Figure 8C).

### Verification of the Relative Expression Level of Feature Genes by qRT–PCR

**FIGURE 9:**
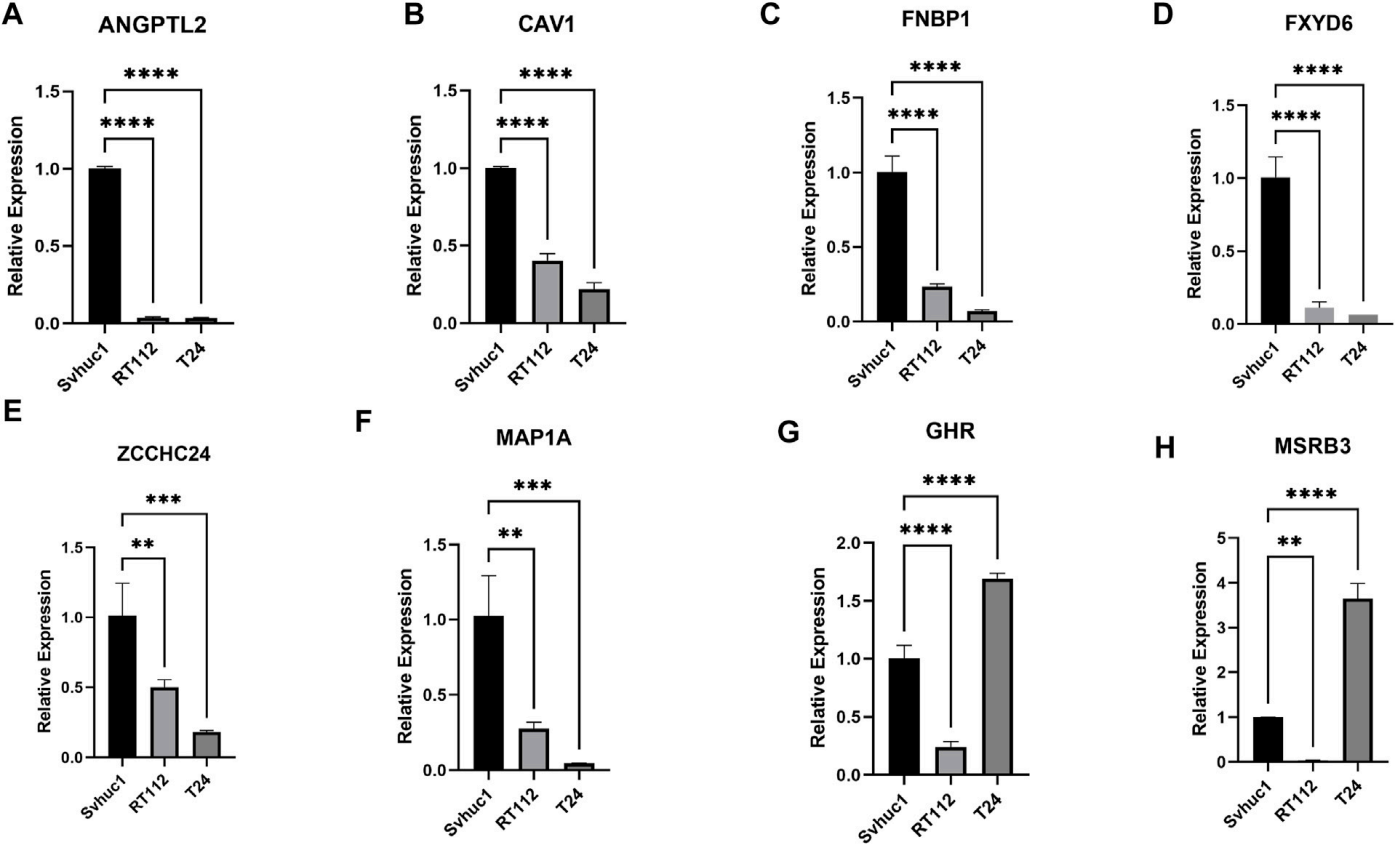
**(A–H)** The relative expression levels of the feature genes in Svhuc-1, RT112, and T24 cells were detected by qRT–PCR. (*p < 0.05, **p < 0.01, ***p < 0.001, ****p < 0.0001).

We adopted qRT–PCR to analyze the relative differential expression of these feature genes *in vitro*. The expression levels of a portion of feature genes, such as ANGPTL2, CAV1, FNBP1, FXD6, MAP1A, and ZCCHC24 were significantly decreased in bladder tumor cell lines (Figures 9A–F). Interestingly, we also found that a small portion of genes was not consistently expressed in different tumor cell lines, such as GHR and MSRB3. Their expression levels were decreased in the RT112 cell line but increased in the T24 cell line (Figures 9G,H).

## Discussion

BLCA ranks ninth among all cancer deaths in China and accounts for 14% of cancer deaths worldwide, although various diagnostic and therapeutic methods have developed rapidly (Liu et al., 2019). Timely and accurate identification of BLCA will hopefully improve therapeutic effects, recurrence rates and patient outcomes (Jordan and Meeks, 2019). Reliable biomarkers for the pristine diagnosis and prognosis assessment of BLCA are profoundly meaningful but still absent (Kim et al., 2014). Identified CDC20 and ASPM as potential immunotherapeutic targets for BLCA, but their study lacked profiling datasets used to verify the screening results Xu et al. (2020). Kong DB et al. constructed a prognostic model for BLCA based on NFAT2, but reliable experiments to verify the model remained to be supplemented (Dai et al., 2021). In our study, we constructed a neural network model containing 14 feature genes to provide novel assistance for the early diagnosis and evaluation of BLCA.

In this study, DEGs were first identified from expression profiling datasets downloaded from the GEO public functional genomics database. Through the visualization of GO and KECG enrichment analyses, we observed that DEGs were markedly associated with muscle system processes and collagen−containing and focal adhesion signaling pathways. Focal adhesions are types of integrin adhesions and are linked to contractile bundles made of F-actin and the motor protein myosin II (Revach et al., 2020). Focal adhesions influence smooth muscle cell contraction and are indispensable for mechanical stability. Focal adhesion signaling contributes to the genesis of a variety of smooth muscle cell phenotypes and has potential implications for mechanical homeostasis beyond calcium mechanisms (Ribeiro-Silva et al., 2021). Multiple prosurvival signaling molecules, such as integrins, growth factor receptors and intracellular molecules, are included in focal adhesion signaling hubs and serve as potential tumor targets (Eke and Cordes, 2015). Verified that focal adhesion kinases crucially regulate TGFβ-induced migration and invasion of BLCA cells Kong et al. (2017). Our algorithm enabled us to filter out 14 feature genes that subsequently were endowed with different scores and worked as input factors to construct a neural network model. These genes included STON1, INA, SGCA, GHR, ANTXR2, ANGPTL2, CAV1, MSRB3, CPXM2, ZCCHC24, MARCKSL1, FNBP1, MAP1A, and FXYD6. Previous studies have identified discrepant biomarkers in proliferating bladder carcinoma cells, and a portion of these feature genes are related to tumorigenesis and development.

MARCKS-related protein (MARCKSL1) is a widespread, highly conserved membrane-associated protein whose hyperexpression promotes cell proliferation via the ErbB2-mediated signalling pathway and facilitates angiogenesis and growth in carcinoma cells *in vivo* (Weimer et al., 2009; Chen et al., 2021). Our study also illustrated the upregulation of MARCKSL1 in BLCA cells. In some cancer contexts, abundant expression of ANGPTL2 is highly related to the frequencies of carcinogenesis and metastasis and shortened survival periods (Endo et al., 2014; Kadomatsu et al., 2014; Gao et al., 2015). A recent study discovered that host ANGPTL2 also shows tumor-suppressive activity by enhancing dendritic cell-mediated CD8^+^ T-cell antitumor immune responses in murine syngeneic models (Horiguchi et al., 2021). In our study, we found that the expression levels of ANGPTL2 in T24 and RT112 cells were lower than those in normal bladder urothelium Svhuc-1 cells. Established animal models have demonstrated that mice lacking SGCA developed cancer-associated mutation of p53 and mutation or altered splicing of Mdm2 (Fernandez et al., 2010). CAV1 is an integral membrane that works not only as a tumor promotor but also as a suppressor (Carver and Schnitzer, 2003). It has been reported that low expression of CAV1 favors tumor progression by promoting cell proliferation, angiogenesis, and metastasis, although re-expression of CAV1 can be detected in later tumor stages (Senetta et al., 2013; Nwosu et al., 2016; Ketteler and Klein, 2018). Low CAV1 expression levels in bladder transitional carcinoma cells were detected in our PCR experiments.

The GHR signaling pathway plays a huge role in growth, metabolism, cell cycle control, and immunity, and its dysfunction enhances the sensitivity to sorafenib through the inactivation of the PI3K/AKT/ERK1/2 signaling pathway (Gao et al., 2020; Strous et al., 2020). MsrB3 is a protein repair enzyme that acts as an antioxidant to eliminate cellular reactive oxygen species (Lee et al., 2014). Concluded that MsrB3 deficiency contributed to the downregulation of p53 and the disturbance of calcium homeostasis in cancer cells Kwak and Kim (2017). Interestingly, we found that the two abovementioned genes were expressed at lower levels in RT112 cells and higher levels in T24 cells. The contradictory expression profiles could be understood for the following reasons: the tumor cells were at different stages of growth, the tumor cells originated from different positions or the tumor cells varied in degree of malignancy. Generally, the results of our experiments are in accordance with previous studies and further enhance the reliability of the model.

Furthermore, increasing evidence has linked cancer to inflammation and immune system activation, and immune-related genes can potentially carry prognostic and therapeutic value (Soria and Shariat, 2018; Soria et al., 2019). T follicular helper cells (Tfh) characteristically express CXCR5 and can provide the critical function of B-cell help (Vinuesa et al., 2016). Tfh cells are strongly associated with B cells and promote antitumor CD8 T-cell responses through the B cell-TFH cell-IL21 axis (Cui et al., 2021). B cells promote the differentiation of tumor-specific CD4 Tfh cells depending on neoantigens, strengthen CD8 T-cell effector abilities by producing IL-21 and finally promote antitumor immunity. We discovered a high content of Tfh cells in bladder tumor tissues, as previous studies have illustrated. Tfh cells have the potential to serve as a novel prognostic and immunotherapeutic signature that could guide clinical management and personalized immunotherapy.

Considering that not all feature genes were linked to the pathological process of BLCA, further validation of the model was our follow-up research step. To calculate the reliability of our model, a ROC curve was drawn to present the specificity and sensitivity of the neural network model, and its AUC value was 0.950 (95% CI: 0.850–1.000). In addition, GSE100926 was subjected to the same procedures for further verification of the predictive accuracy of the model, and the ultimate AUC value was 0.667 (95% CI: 0.667–1.000).

In addition, several previous studies contributed to predicting BLCA using artificial intelligence and machine learning algorithms with gene expression profiling. Applied genetic programming algorithm to evolve classifier mathematical models including 21 genes for outcome prediction Bartsch et al. (2016). The Random Forest algorithm was formulated by Breiman (2001) and the random forest was an ensemble of multiple decision trees. Based on decision trees, a Random Forest can serve as a classifier by aggregating individual tree predictors which have been built using randomly sampled bootstrap observations from the original data and provide valuable information on variable importance (Sampson et al., 2011). The advantages of the random forest included good performance, little tuning and variable importance measures, which make the algorithm a powerful tool with appealing characteristics for use on quantitative bioinformatical data (Epifanio, 2017). In our study, the smaller number of key genes and the implementation of a random forest algorithm were the unique advantages.

Nevertheless, there were some limitations in our study. The experimental genomics data were all downloaded from the GEO database. Single data sources might somewhat limit the effectiveness of forecasting operations, and sufficient validation datasets from another unrelative database were necessarily replenished. In addition, not all feature genes had reliable research to substantiate their association with tumorigenesis development in BLCA. The lack of external validation of molecular experiments might also be the flaw of our study.

## Conclusion

We constructed a 14-feature-gene-based neural network model to furnish novel diagnosis and evaluation tools for BLCA. After repeated validation, although our study still needed further verification, the model was found to be competent to make early accurate diagnosis decisions for patients with BLCA.

## Data Availability

The original contributions presented in the study are included in the article/supplementary material, further inquiries can be directed to the corresponding authors.

## Abbreviations

BLCA, Bladder cancer; GEO, Gene Expression Omnibus; GO, Gene Ontology; KEGG, Kyoto Encyclopedia of Genes and Genomes; DEGs, differentially expressed genes; PPI, protein–protein interaction, ROC, receiver operating characteristic; AUC, area under the curve; IRGs, immune-related genes; Tfh, T follicular helper cells.
